# Adrenomedullin Therapy for Moderate-to-Severe COVID-19 Pneumonia: Double-Blind Placebo-Controlled Phase 2a Trial

**DOI:** 10.3390/v17070982

**Published:** 2025-07-14

**Authors:** Toshihiro Kita, Norio Ohmagari, Sho Saito, Hiroshi Mukae, Takahiro Takazono, Taka-Aki Nakada, Tadanaga Shimada, Yuji Hirai, Yuichiro Shindo, Kosaku Komiya, Atsushi Saito, Masaya Yamato, Koichiro Homma, Masaki Okamoto, Yoshihiro Yamamoto, Yoshikazu Mutoh, Chihiro Hasegawa, Nobuaki Mori, Fukumi Nakamura-Uchiyama, Mitsuru Honda, Keisuke Tomii, Hiroshi Ishii, Ichiro Takajo, Koji Watanabe, Kazuo Kitamura

**Affiliations:** 1 Department of Projects Research, Frontier Science Research Center, University of Miyazaki, Miyazaki 889-1692, Japan; 2 Disease Control and Prevention Center, National Center for Global Health and Medicine, Japan Institute for Health Security, Tokyo 162-8655, Japan; 3 Department of Respiratory Medicine, Nagasaki University Graduate School of Biomedical Sciences, Nagasaki 852-8520, Japan; 4 Department of Emergency and Critical Care Medicine, Chiba University Graduate School of Medicine, Chiba 260-8670, Japan; 5 Department of Infectious Diseases, Tokyo Medical University Hachioji Medical Center, Hachioji 193-0998, Japan; 6 Department of Respiratory Medicine, Nagoya University Graduate School of Medicine, Nagoya 466-8550, Japan; 7 Respiratory Medicine and Infectious Diseases, Oita University Faculty of Medicine, Oita 879-5593, Japan; 8 Department of Respiratory Medicine and Allergology, Sapporo Medical University School of Medicine, Sapporo 060-8556, Japan; 9 Department of General Internal Medicine and Infectious Diseases, Rinku General Medical Center, Izumisano 598-8577, Japan; 10 Department of Emergency and Critical Care Medicine, Keio University School of Medicine, Tokyo 160-8582, Japan; 11 Department of Respirology, NHO Kyushu Medical Center, Fukuoka 810-0065, Japan; 12 Department of Clinical Infectious Diseases, Graduate School of Medicine and Pharmaceutical Sciences, University of Toyama, Toyama 930-8555, Japan; 13 Department of Infectious Diseases, Tosei General Hospital, Seto 489-8642, Japan; 14 Nagoya City University East Medical Center, Nagoya 464-8547, Japan; 15 Department of General Internal Medicine and Infectious Diseases, NHO Tokyo Medical Center, Tokyo 152-8902, Japan; 16 Department of Infectious Diseases, Tokyo Metropolitan Bokutoh Hospital, Tokyo 130-8575, Japan; 17 Critical Care Center, Toho University Omori Medical Center, Tokyo 143-8540, Japan; 18 Department of Respiratory Medicine, Kobe City Medical Center General Hospital, Kobe 650-0047, Japan; 19 Department of Respiratory Medicine, Fukuoka University Chikushi Hospital, Fukuoka 818-8502, Japan; 20 Division of Respirology, Rheumatology, Infectious Diseases, and Neurology, Department of Internal Medicine, Faculty of Medicine, University of Miyazaki, Miyazaki 889-2192, Japan

**Keywords:** adrenomedullin, COVID-19, pneumonia, mechanical ventilation, phase 2a clinical trial, Japanese

## Abstract

Adrenomedullin (AM) is a bioactive peptide that is strongly induced during severe inflammation, including pneumonia and sepsis, and serves as an organ-protective factor. The plasma concentration of AM is markedly increased in the novel coronavirus disease COVID-19 and is closely related to the severity of the disease and prognosis of patients. We performed two investigator-initiated trials to evaluate the efficacy and safety of AM in patients with moderate-to-severe COVID-19. This multicenter, double-blind, placebo-controlled phase-2a trial evaluated COVID-19 patients with severe (*n* = 33) and moderate (*n* = 31) pneumonia in Japan. Patients were randomly assigned to receive either 15 ng/kg/min AM or placebo. The primary endpoint was the duration of mechanical ventilation (MV) for severe pneumonia and oxygen support for moderate pneumonia. The main secondary endpoint was clinical status up to 30 days after the intervention. No differences in primary or secondary endpoints were observed between the AM and placebo groups in patients with severe or moderate pneumonia. In the severe pneumonia group, three patients in the placebo group died due to respiratory failure, and one patient in the AM group died due to respiratory failure. The respiratory function test at 30 days in the moderate pneumonia group tended to be better than that in the AM group and approached significance (*p* = 0.073). Although mild adverse events caused by the vasodilatory effects of AM were noted, the safety of AM for treating pneumonia was confirmed. In these trials, we did not observe a definitive efficacy of AM in moderate to severe pneumonia. Alternative strategies for the treatment of AM in pneumonia require further research.

## 1. Introduction

Severe acute respiratory syndrome coronavirus 2 (SARS-CoV-2) causes a novel coronavirus disease (COVID-19) that includes various levels of pneumonia. The cumulative impact of the COVID-19 pandemic is serious; millions of patients have died from severe respiratory failure, with 7.1 million deaths in 778 million cases as of April 2025 [1]. Therapeutic methods for severe pneumonia caused by COVID-19, especially for those who required mechanical ventilation, were limited, and a significant proportion of patients died in the early phase of the pandemic. We initiated the first clinical trial in this phase of the pandemic using adrenomedullin (AM) to rescue critically ill patients through alternative pathways besides antiviral drugs or immunosuppressive agents [2]. Subsequently, we extended AM treatment for patients with moderate pneumonia to treat patients before severe conditions.

AM is an endogenous bioactive peptide with strong vasodilative activity that regulates blood circulation and is mainly produced in vascular endothelial cells [3]. AM exhibits various biological activities, including immunological modulation [2]. The most prominent increase in the plasma concentration of AM has been observed in severe inflammatory diseases, such as sepsis and severe pneumonia in COVID-19 [4,5]. More significantly, increased plasma AM levels in severe diseases are closely associated with mortality and severe clinical events [6]. These data strongly suggest that AM should work as a crucial factor in severe inflammatory status, friend or foe aside. In experimental septic models, tissue damage was exceptionally worsened in mice with partially suppressed AM expression, and conversely, tissue damage was well suppressed in mice overexpressing AM; therefore, AM is thought to work as a tissue-protective factor in severe inflammation [7,8]. As expected, the exogenous administration of AM ameliorated organ damage and mortality in experimental septic models [9,10]. Müller–Redetzky et al. reported that AM may be effective for ventilator-associated organ damage such as ventilator-induced lung injury (VILI) [11]. Inspired by these data, we planned the therapeutic application of AM for severe pneumonia with mechanical ventilation caused by COVID-19. AM can improve both inflammatory tissue damage and VILI in severe pneumonia, and decrease patient mortality. We previously developed an AM formulation and completed phase 1 and 2a trials for inflammatory bowel disease (IBD) [12,13,14]. We initially planned a similar administration program for COVID-19 as that for IBD, based on the results of the trials. However, we increased the administration dosage to contend with the most severe condition by introducing a 72 h continuous infusion of AM [2]. In this multicenter, randomized, double-blind, placebo-controlled study, we evaluated the efficacy and safety of AM in Japanese patients with moderate-to-severe pneumonia caused by COVID-19.

## 2. Materials and Methods

### 2.1. Study Design

The present trials were randomized, double-blind, multicenter, placebo-controlled, phase 2a studies. Fifteen medical centers for the severe pneumonia trial and 21 medical centers for the moderate pneumonia trial in Japan participated in this study. After obtaining approval from the Pharmacological and Medical Device Agency, ethical approval for this study was obtained from the institutional review boards of the University of Miyazaki and other centers. This clinical trial was conducted in compliance with the ethical principles of the Declaration of Helsinki, Good Clinical Practice (GCP) of the Japanese Ministerial Ordinance, and other related regulations. The trials were registered with the Japan Registry of Clinical Trials (jRCT2071200041 and jRCT2071210038).

### 2.2. Subjects

In the severe pneumonia trial, eligible subjects were confirmed to be COVID-19 patients aged 20–75 years who required mechanical ventilation for respiratory failure. However, patients with extracorporeal membrane oxygenation (ECMO) were not eligible. Patients with renal failure (serum creatinine > 2.0 mg/dL), liver damage (AST and/or ALT aminotransferase > 5 times the standard values), or a past or current history of malignancy were excluded. Patients with significant ECG abnormalities or a history of heart failure, myocardial infarction, or angina pectoris were excluded from the study.

In the moderate pneumonia trial, eligible subjects were confirmed to be COVID-19 patients aged 20–85 years, who required oxygen support for respiratory failure within 10 days of symptom appearance. The means for oxygen support included a nasal cannula, oxygen mask, non-invasive ventilation, and high-flow oxygen therapy; however, mechanical ventilation and ECMO were not eligible. The exclusion criteria were the same as those used in the trial for severe pneumonia. In addition, patients with respiratory failure before COVID-19 onset were excluded from the study. All patients or their proxies provided written informed consent for all study-related procedures. The target numbers of patients were 40 for severe pneumonia and 60 for moderate pneumonia.

### 2.3. Randomization and Masking

Eligible patients were enrolled by the principal investigator or designer based on the inclusion and exclusion criteria. Patients were randomized into one of two groups at a ratio of 2:2 to receive a placebo or 15 ng/kg/min AM, which was the highest safety-confirmed dose in a phase 1 trial [12]. Randomization was performed by an independent contract research organization, CAC Croit (Sapporo, Japan; the company name was changed to EP Croit and then EPS), using a block size of four. The institute then adjusted the allocations.

### 2.4. Interventions

We prepared three AM formulations for the trials, namely huAM-003, huAM-004, and huAM-005. The limited expiration date of the formulations and expansion of the number of institutes required additional formulations. The formulations were prepared from the same bulk AM using an established manufacturing process at a pharmaceutical company (Fuji Yakuhin, Toyama, Japan), and the quality of the formulations was strictly controlled. No differences between the formulations were observed in the confirmatory analyses. The contents of the formulations were the same as in previous trials for IBD and the patients were hospitalized before the eligibility evaluation [13,14]. All drugs for COVID-19, including antiviral drugs currently permitted by the Japanese Ministry of Health and Welfare, were allowed as needed. The patients received continuous infusion of the assigned drug, namely AM or placebo, for 72 h and then received intermittent administration of the drug for 8 h per day for up to 7 days. In the severe pneumonia trial, test drug administration must be initiated within 24 h after the introduction of mechanical ventilation. The trial drug administration was terminated when the patient withdrew from mechanical ventilation (severe pneumonia) or oxygen support (moderate pneumonia). A detailed schema of the study design was provided in a previous paper [2]. The clinical status of patients was monitored for 30 days after the initiation of drug administration. Patients with moderate pneumonia were encouraged to visit the hospital for a respiratory function test (RFT) 30 days after the beginning of intervention.

### 2.5. Data Assessment

All data were collected at each institute from November 2020 to October 2021 for severe pneumonia, and from July 2021 to April 2022 for moderate pneumonia. The primary endpoints were the durations of mechanical ventilation for severe pneumonia and oxygen support for moderate pneumonia after the beginning of the intervention. The mutual major secondary endpoints were the clinical six-category ordinary scores up to 30 days after the initiation of the test drug. The six-category ordinary scores consisted of the following categories: (1) discharged with recovery; (2) hospitalized, not requiring oxygen support; (3) hospitalized, requiring oxygen support; (4) hospitalized, requiring nasal high-flow oxygen therapy, noninvasive mechanical ventilation, or both; (5) hospitalized, requiring invasive mechanical ventilation or ECMO; and (6) death. Ordinary scores were assessed at 15 and 30 days after the beginning of the intervention for severe pneumonia and at 10, 20, and 30 days after the beginning of the intervention for moderate pneumonia. Almost all patients were expected to recover within 20 days. In the trial for moderate pneumonia, RFT, including spirogram and carbon monoxide diffusion capacity (DLco), was performed 30 days after the intervention. Plasma concentrations of inflammatory-related cytokines and other biomarkers including interleukin (IL)-1β, IL-6, IL-10, tumor necrosis factor (TNF)-α, interferon-γ, autotaxin, fibrin/fibrinogen degradation products (FDP), D-dimer, tissue plasminogen activator (t-PA), plasmin-α2 plasmin inhibitor complex (PIC), and pentraxin3 (PTX3) were assessed before and after intervention. All the measurements were performed using a laboratory testing service provided by SRL (Tokyo, Japan). AM plasma concentrations were not measured in the severe pneumonia trial because of strict restrictions on infected samples from previous institute, where there was no system to handle inexperienced pathogens. Therefore, we established an AM measurement system in accordance with the GCP in cooperation with LSI Medience and other laboratory testing services (Tokyo, Japan) for the moderate pneumonia trial. The serum AM concentration was assessed using an automated enzyme-linked immunosorbent assay (ELISA) system (AIA-1800, Toso, Tokyo, Japan). Blood sampling and the procedure for measuring AM concentration were previously described [12].

Safety evaluations, including the evaluation of adverse events (AEs) and serious adverse events (SAEs), were conducted throughout the study. Vital signs, blood and urine tests, and 12-lead ECG were performed at specific time points after drug administration.

### 2.6. Data Collection and Statistical Analysis

All data were collected using an electronic data collection system (cubeCDMS) and analyzed by Intellim (Tokyo, Japan), an independent contract research organization. For the severe pneumonia trial, based on 80% power, a level of 0.05, 11 days as the expected duration of mechanical ventilation in the placebo group, and a reduction of 4 days by AM treatment, a total sample size of 32 patients was required. Similarly, for the moderate pneumonia trial, based on 10 days for the expected duration of oxygen support in the placebo group and a reduction of 4 days by AM treatment with larger dispersion, a total sample size of 50 was required. Therefore, we decided on a sample size of 40 for severe pneumonia and 60 for moderate pneumonia with a margin. The magnitude of the AM effect was selected because a meaningful effect would be required for clinical importance, warranting further study. Primary endpoints, that is, the duration of mechanical ventilation or oxygen support, were analyzed using the Kaplan–Meier method with a log-rank test. Additionally, the unpaired *t*-test or Wilcoxon signed-rank test was used after confirmation of normality using the Shapiro–Wilk test. Clinical status and six-category ordinary scores were analyzed using the Wilcoxon signed-rank test. Changes in biomarkers that were logarithmically transformed as needed and parameters in RFT were analyzed using an unpaired *t*-test. The significance level for each test was set at 5%. All analyses were performed using SAS version 9.4 (SAS Institute, Cary, NC, USA) and SPSS Ver. 27 (IBM Corp., Armonk, NY, USA). All data are presented as mean ± standard deviation (SD).

## 3. Results

### 3.1. Randomization and Clinical Characteristics of Patients at Baseline

**Figure 1:**
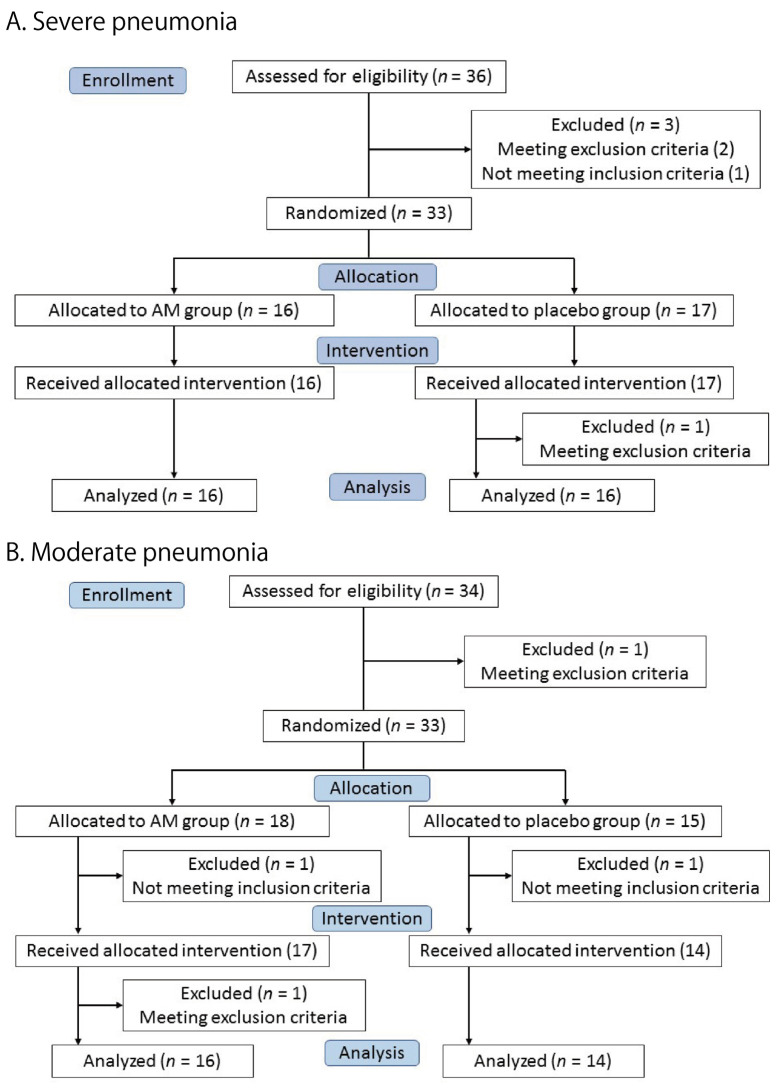
Flow diagram of the two trials.

Thirty-three patients were enrolled in the severe pneumonia trial. A total of 16 patients were allocated to the AM group and 17 patients to the placebo group (Figure 1A). All patients received the allocated test drug, but one patient in the placebo group was excluded because of a violation of the exclusion criteria (ECG abnormality). Finally, 16 patients in each group were included in the full primary analysis set (FAS).

Figure 1B shows the moderate pneumonia trial. Eighteen and fifteen patients were allocated to the AM and placebo groups, respectively. One patient in each group was excluded before the intervention. A total of 17 patients in the AM group and 14 patients in the placebo group received the test drugs. One patient in the AM group was excluded after the intervention due to violation of the exclusion criteria (ECG abnormality). The remaining 16 patients in the AM group and 14 in the placebo group were included in the primary FAS.

All patients who received the test drugs in both the trials were included in the safety analysis. The clinical characteristics of the patients are summarized in Table 1. No significant differences were observed between the AM and placebo groups in either trial. At that time, the only available antiviral drug was remdesivir, and almost all patients received this drug. The best supportive care, including steroids and anticoagulants, was provided to the patients.

### 3.2. Clinical Efficacy

**Figure 2:**
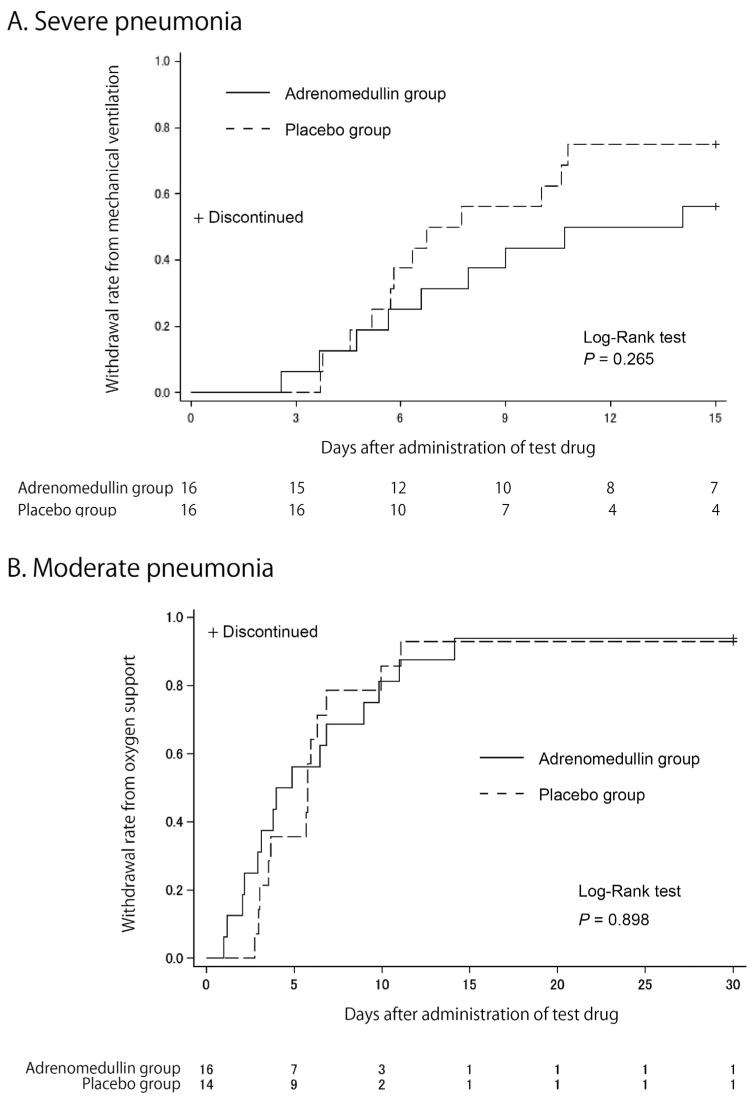
Kaplan–Meier estimates of cumulative recovers. Cumulative recovery estimates are shown in patients with severe pneumonia (**A**) and in patients with moderate pneumonia (**B**).

As shown by the Kaplan–Meier curves in Figure 2, we did not observe any differences in the primary endpoint between the two groups in either trial. The mean durations for mechanical ventilation in severe pneumonia were 10.6 ± 4.8 days and 7.8 ± 4.3 days for the AM and placebo groups, respectively (*p* = 0.346, Wilcoxon signed-rank test). The mean durations for oxygen support in moderate pneumonia were 7.0 ± 7.2 days and 7.4 ± 7.0 days for the AM and placebo groups, respectively (*p* = 0.618, Wilcoxon signed-rank test).

**Figure 3:**
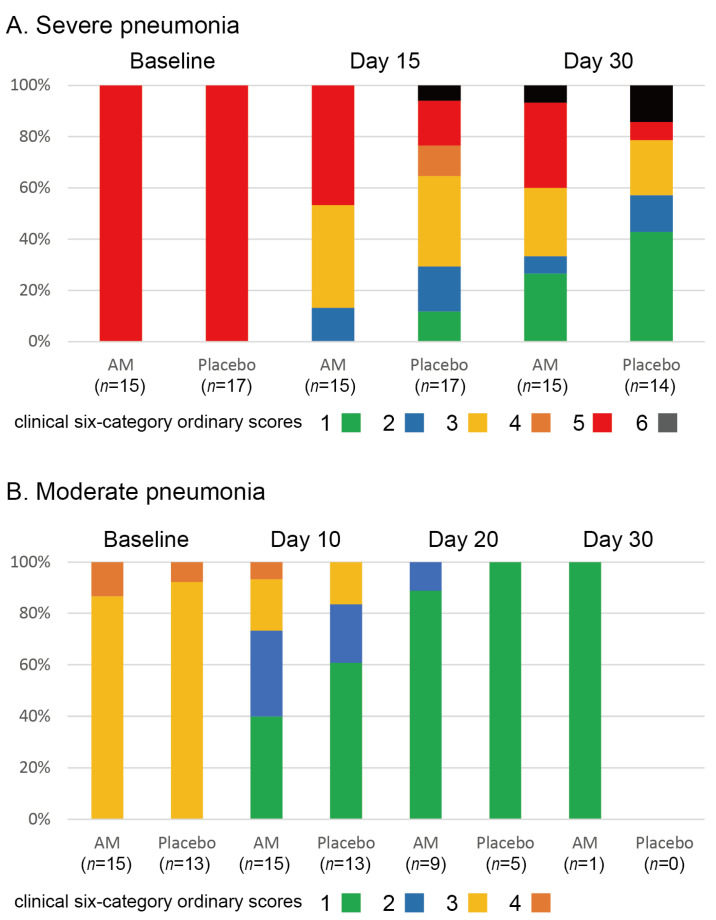
Stacked bar chart for clinical 6-category ordinary scores in two trials.

The stacked bar chart for the clinical six-category ordinary scores is shown in Figure 3. We found no differences in the clinical status of severe pneumonia (*p* = 0.226 at 15 days and *p* = 0.237 at 30 days, Wilcoxon signed-rank test). In addition, no differences were observed in changes in clinical status in moderate pneumonia (*p* = 0.26 at 10 days, *p* = 0.39 at 20 days, and *p* = 1.00 at 30 days, Wilcoxon signed-rank test). Three patients in the placebo group and one patient in the AM group died of respiratory failure. The survival rate at 30 days in the placebo group was 0.813, and that in the AM group was 0.933. No significant difference between the two groups was observed (*p* = 0.338, Kaplan–Meier test).

Furthermore, we found no significant differences in the biomarker changes (Table 2 and Table 3).

The RFT results for patients with moderate pneumonia at 30 days are summarized in Table 4. This test was performed after the discharge of all patients; thus, the participants were limited. The AM group showed favorable values for all parameters, although the differences were not statistically significant.

### 3.3. AM Plasma Concentrations

**Figure 4:**
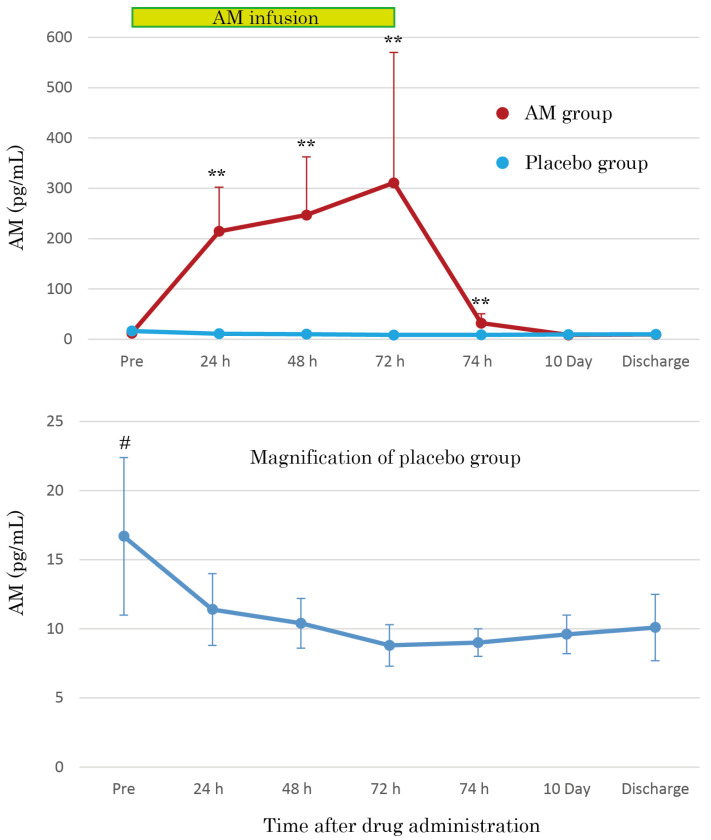
Mean plasma concentration–time profiles of AM during 72 h continuous administration of AM in patients with moderate pneumonia. Additionally, the mean plasma concentrations of AM at 10 days and at the discharge point are illustrated. The magnification of the placebo group shows the natural course of plasma AM concentration in moderate pneumonia. ** *p* < 0.01, compared to basal value. # *p* < 0.05, compared to other points of the course.

The AM plasma concentrations were dramatically increased by continuous AM infusion, reaching over 30 times those of the placebo group after 72 h (Figure 4). The increased AM plasma concentrations mostly returned to basal levels 2 h after terminating AM infusion, but the concentrations remained at significantly increased levels. As shown in the magnification of the placebo group in Figure 4, the AM plasma concentrations gradually decreased over time. Furthermore, the pre-infusion AM concentration values were significantly higher than those at other time points (*p* < 0.01).

### 3.4. Safety Assessments

All patients who received the test drugs were included in safety assessments. Patients excluded from the FAS because of violations of the exclusion criteria, namely, one patient in the placebo group in the severe pneumonia trial and one patient in the AM group in the moderate pneumonia trial, were included. The reported AEs are listed in Table 5. Because of the severity of the disease, many AEs were reported in almost all patients in these trials. One patient in the AM group and three patients in the placebo group in the severe pneumonia trial died of respiratory failure due to COVID-19. One patient in the AM group in the moderate pneumonia trial died of respiratory failure; however, this patient was excluded from the FAS. All SAEs in both trials were determined as COVID-19-related disease states by the principal investigators. AM has a vasodilative effect; therefore, the AM group showed more frequent blood pressure reduction than the placebo group. Three patients in the AM group and one patient in the placebo group were discontinued from administration of the test drug because of blood pressure reduction in the trial for severe pneumonia. Blood pressure reduction also occurred in the AM group in the moderate pneumonia trial, but the extent remained within the allowable range. Nine patients in the AM group and seven patients in the placebo group (trial for severe pneumonia), and ten patients in the AM group and five patients in the placebo group (trial for moderate pneumonia) reported side effects. All side effects were mild or moderate, and no serious or lethal side effects were reported in either trial.

## 4. Discussion

This is the first (to our knowledge) randomized placebo-controlled phase 2a trial of AM in Japanese patients with moderate-to-severe pneumonia caused by COVID-19. Unfortunately, we could not find any benefits of AM for COVID-19 pneumonia at either the primary or secondary endpoints. The main purpose of the trials was to shorten the treatment period of pneumonia by AM administration; however, contrary to our expectations, the resulting primary endpoint and clinical status transition may suggest a prolongation of the treatment period by AM administration (Figure 2 and Figure 3). This was an unfortunate result; however, the beneficial potential of AM remains. Fewer deaths in the late phase of treatment in severe pneumonia (Figure 3) and better respiratory function on day 30 of treatment in moderate pneumonia (Table 4) might suggest better tissue repair after AM administration, as suggested in trials for IBD [13,14].

Another crucial factor in this weak result was the shortage of patients in the target number. In the severe pneumonia trial, 32 patients had FAS, which was 80% of the target number. Moreover, the number of patients with FAS in moderate pneumonia was 30 for a target number of 60, and the achievement ratio was only 50%. It is possible that a sufficient number of patients might produce favorable results. Conversely, these low achievement ratios reveal the essential problem of trials, in which the trial strategy may not match the actual demands of clinical settings. The impact of the transition of SARS-CoV-2 variants is crucial for these trials. The trial for severe pneumonia was terminated by the end of the delta variant prevalence and did not receive a major influence from the variant. However, the trial for moderate pneumonia began with delta variant prevalence and stopped with omicron variant prevalence. The omicron variant exhibited lower in-hospital mortality and respiratory-failure rates than the delta variant [15]. Decreased infectivity in the lung and lower pathogenicity of the omicron variant [16] introduced a decrease in primary viral pneumonia but increased complicated pneumonia, such as aspiration pneumonia, in older patients [17]. This change in pathology caused serious problems in our trial. The main target of the trial, namely patients with pure viral pneumonia in moderate-to-severe conditions, could not be found in the central medical facilities for COVID-19. Instead, patients of older age and those with complicated pneumonia and other complications such as cardiac failure were included in our trial facilities. As a result, the trial for moderate pneumonia was completely stalled by the prevalence of the omicron variant, despite the best promotion for the trial. Alternative targets for pneumonia therapy, such as the reduction of prolonged tissue damage to the lungs, will be required in future trials.

The safety of AM formulations has been confirmed in phase 1 and phase 2 trials for IBD [10,11,12]. Recently, its safety profile was validated in patients with acute cerebral infarction [18]. In these trials, the vasodilative properties of AM affected the hemodynamic state of patients. However, a specific treatment for this effect, namely a vasopressor or additional infusion, has not been reported; therefore, appropriate monitoring of hemodynamics and adequate withdrawal of AM infusion, if needed, will enable the utilization of AM even in severe conditions caused by COVID-19. In particular, we attempted a 72 h continuous AM infusion for the first phase of treatment to maximize the effect of AM against severe conditions in patients. The AM plasma concentration reached approximately 300 pg/mL 72 h after AM administration (Figure 4). The AM plasma levels were significantly higher than those in previous trials and reached comparable levels in patients with severe conditions, such as ARDS or sepsis [4]. Proof of safety at the highest levels ever reached is crucial for extending AM treatment in the future.

## 5. Conclusions

The current trials revealed the general safety of continuous administration of high-dose AM for 72 h in critically ill patients. This result will be useful for expanding the application of AM to other disease fields. Unfortunately, we were unable to demonstrate the efficacy of AM in moderate-to-severe COVID-19 pneumonia. Alternative strategies for the treatment of AM in pneumonia require further research.

## Figures and Tables

**Table 1:** Basal characteristics of the patients.

Trial		Severe Pneumonia			Moderate Pneumonia		
Group		AM	Placebo		AM	Placebo	
Patient		N = 16	N = 16	*p* Value	N = 16	N = 14	*p* Value
Demographic statistics	Age (years)	61.3 ± 9.9	58.3 ± 8.0	0.35	53.4 ± 13.5	47.6 ± 14.1	0.26
	BMI	27.5 ± 5.5	29.0 ± 5.2	0.46	27.2 ± 5.1	27.9 ± 4.0	0.71
	Sex (male)	13 (81.3%)	13 (81.3%)	―	10 (62.5%)	12 (85.7%)	0.29
	Smoking	7 (43.8%)	9 (56.3%)	0.56	7 (43.8%)	8 (57.1%)	0.55
Complication(s)	Any	15 (93.8%)	16 (100%)	―	14 (87.5%)	12 (85.7%)	0.95
	Diabetes	5 (31.3%)	8 (50%)	0.38	2 (12.5%)	1 (7.1%)	0.82
	Hypertension	7 (43.8%)	11 (68.8%)	0.24	6 (37.5%)	4 (28.6%)	0.70
Vaccination	Once/Twice	―	―		0/4	1/2	
Clinical severity	SOFA	4.9 ± 2.6	5.3 ± 2.5	0.68	2.3 ± 1.9	1.9 ± 0.8	0.33
	APACHE II	12.0 ± 7.1	12.1 ± 6.5	0.98	―	―	
Laboratory test	IL-6 (pg/mL)	467 ± 878	263 ± 371	0.40	54 ± 147	65 ± 112	0.83
	D-dimer (mg/mL)	6.8 ± 11.2	3.2 ± 5.5	0.26	1.4 ± 2.4	1.0 ± 0.4	0.55
	PTX-3 (ng/mL)	56.5 ± 44.9	81.7 ± 95.5	0.35	53.0 ± 53.6	61.3 ± 45.7	0.66
	CRP (mg/dL)	5.1 ± 3.3	10.2 ± 9.6	0.054	6.1 ± 3.6	9.8 ± 7.5	0.11
Concomitant drug	Remdesivir	13 (81.3%)	16 (100%)	0.38	15 (93.8%)	13 (92.9%)	―
	Steroid	16 (100%)	16 (100%)	―	15 (93.8%)	14 (100%)	0.79
	Anticoagulants	15 (93.8%)	15 (93.8%)	―	9 (56.3%)	8 (57.1%)	―
	Immunomodulator	8 (50%)	10 (62.5%)	0.56	7 (43.8%)	8 (57.1%)	0.55

**Table 2:** Inflammatory cytokines and related parameters in severe pneumonia.

			Before Treatment			After Continuous Infusion			After Intermittent Infusion	
	Group	AM	Placebo	*p* Value	AM	Placebo	*p* Value	AM	Placebo	*p* Value
	N	16	16		9	14		15	16	
IL-1b	pg/mL	0.9 ± 3.8	0 ± 0	0.33	0 ± 0	0 ± 0	―	0.9 ± 3.6	0.9 ± 3.8	0.998
IL-6	pg/mL	467 ± 878	263 ± 371	0.40	802 ± 1783	729 ± 2016	0.93	578 ± 1524	1804 ± 4114	0.29
IL-8	pg/mL	19.0 ± 21.9	13.5 ± 7.1	0.35	10.3 ± 8.6	12.9 ± 19.0	0.71	9.5 ± 16.0	18.1 ± 36.0	0.41
IL-10	pg/mL	7.6 ± 11.9	10.9 ± 14.8	0.49	4.6 ± 5.6	1.3 ± 2.8	0.07	1.3 ± 3.8	1.4 ± 4.0	0.94
TNF-a	pg/mL	1.78 ± 1.07	1.89 ± 0.82	0.75	1.79 ± 0.91	2.31 ± 4.37	0.73	2.05 ± 1.81	2.40 ± 2.26	0.64
INF-g	IU/mL	0.11 ± 0.21	0.14 ± 0.28	0.67	0 ± 0	0.20 ± 0.69	0.40	0.03 ± 0.07	0.01 ± 0.05	0.52
ATX	mg/L	0.48 ± 0.20	0.62 ± 0.48	0.30	0.55 ± 0.29	0.61 ± 0.32	0.68	0.51 ± 0.25	0.60 ± 0.30	0.36
FDP	mg/mL	26.4 ± 50.4	9.9 ± 7.9	0.21	6.7 ± 2.1	26.1 ± 43.8	0.20	20.5 ± 37.2	24.4 ± 25.8	0.74
D-dimer	mg/mL	6.77 ± 11.15	3.22 ± 5.51	0.26	2.67 ± 1.66	7.69 ± 9.49	0.13	7.22 ± 10.11	10.61 ± 11.23	0.39
tPA	U/L	183 ± 125	166 ± 151	0.74	162 ± 169	163 ± 160	0.98	145 ± 107	227 ± 354	0.41
PIC	mg/mL	2.38 ± 3.27	1.76 ± 1.14	0.48	0.87 ± 0.50	1.81 ± 1.33	0.055	2.01 ± 2.08	2.21 ± 2.50	0.81
PTX3	ng/mL	56.5 ± 44.9	81.7 ± 95.5	0.35	69.6 ± 58.4	36.3 ± 42.5	0.13	70.7 ± 60.6	31.6 ± 44.8	0.049

**Table 3:** Inflammatory cytokines and related parameters in moderate pneumonia.

			Before Treatment			After Continuous Infusion			After Intermittent Infusion	
	Group	AM	Placebo	*p* Value	AM	Placebo	*p* Value	AM	Placebo	*p* Value
	N	16	14		11	9		15	13	
IL-1b	pg/mL	1.3 ± 3.4	2.4 ± 4.9	0.45	0.9 ± 3.0	2.2 ± 4.4	0.44	2.2 ± 6.3	2.4 ± 4.5	0.93
IL-6	pg/mL	53.8 ± 147.2	64.5 ± 112.4	0.83	58.1 ± 146.6	19.1 ± 30.1	0.45	15.0 ± 36.0	14.4 ± 30.8	0.96
IL-8	pg/mL	8.1 ± 7.1	9.3 ± 7.9	0.68	3.2 ± 5.2	3.4 ± 3.0	0.91	3.0 ± 2.9	2.4 ± 2.5	0.57
IL-10	pg/mL	15.3 ± 27.7	10.3 ± 13.6	0.55	0.2 ± 0.6	0.4 ± 0.9	0.44	0.1 ± 0.5	0.6 ± 1.3	0.19
TNF-a	pg/mL	1.51 ± 0.82	1.50 ± 0.63	0.96	1.11 ± 0.56	1.10 ± 0.33	0.96	1.32 ± 0.59	1.11 ± 0.26	0.25
INF-g	IU/mL	0.46 ± 1.30	0.19 ± 0.24	0.44	0.01 ± 0.03	0.02 ± 0.04	0.44	0.01 ± 0.03	0.02 ± 0.04	0.48
ATX	mg/L	0.57 ± 0.20	0.61 ± 0.29	0.69	0.63 ± 0.24	0.51 ± 0.20	0.23	0.62 ± 0.21	0.56 ± 0.31	0.57
FDP	mg/mL	5.4 ± 3.7	5.6 ± 2.1	0.86	4.7 ± 5.1	5.1 ± 3.0	0.84	4.7 ± 1.5	5.0 ± 2.6	0.68
D-dimer	mg/mL	1.44 ± 2.45	1.03 ± 0.42	0.55	1.92 ± 3.53	1.66 ± 0.99	0.84	1.38 ± 1.21	1.79 ± 1.40	0.42
tPA	U/L	77 ± 64	123 ± 148	0.27	141 ± 125	76 ± 32	0.15	146 ± 300	91 ± 72	0.53
PIC	mg/mL	1.80 ± 0.64	2.10 ± 0.81	0.27	1.21 ± 0.37	1.38 ± 0.47	0.38	1.33 ± 0.50	1.44 ± 0.46	0.54
PTX3	ng/mL	53.0 ± 53.6	61.3 ± 45.7	0.66	21.1 ± 18.8	15.1 ± 12.9	0.43	10.6 ± 17.6	13.6 ± 23.4	0.70

**Table 4:** Respiratory function test.

	Adrenomedullin Group		Placebo Group		
	Actual Value/Standard Value (%)	N	Actual Value/Standard Value (%)	N	*p* Value
Vital capacity	85.7 ± 20.0	11	77.7 ± 10.2	7	0.34
Forced expiratory volume in 1 s	85.9 ± 13.4	11	74.4 ± 10.3	7	0.073
Pulmonary diffusing capacity (DLco)	77.5 ± 14.8	9	67.1 ± 13.3	7	0.15

**Table 5:** Adverse events summary.

Trial	Severe Pneumonia		Moderate Pneumonia	
Group	AM	Placebo	AM	Placebo
No of Cases	N = 16	N = 17	N = 17	N = 14
Any adverse events	15 (93.8%)	16 (94.1%)	14 (82.4%)	10 (71.4%)
Serious adverse events	3 (18.8%)	4 (23.5%)	1 (5.9%)	1 (7.1%)
Death	1 (6.3%)	3 (17.6%)	1 (5.9%)	0
AE occurring in >10%				
Sepsis	3 (18.8%)	2 (11.8%)	―	―
Bacterial pneumonia	2 (12.5%)	3 (17.6%)	2 (11.8%)	0
Delirium	1 (6.3%)	4 (23.5%)	―	―
Insomnia	2 (12.5%)	1 (5.9%)	1 (5.9%)	1 (7.1%)
Headache	0	1 (5.9%)	4 (23.5%)	0
Bradycardia	0	2 (11.8%)	0	1 (7.1%)
Hypertension	2 (12.5%)	1 (5.9%)	―	―
Aspiration pneumonia	1 (6.3%)	2 (11.8%)	―	―
Respiratory failure	1 (6.3%)	3 (17.6%)	―	―
Organized pneumonia	2 (12.5%)	1 (5.9%)	0	1 (7.1%)
Constipation	4 (25.0%)	3 (17.6%)	2 (11.8%)	2 (14.3%)
Diarrhea	3 (18.8%)	3 (17.6%)	0	1 (7.1%)
Upper gastrointestinal bleeding	2 (12.5%)	0	―	―
Rash	2 (12.5%)	2 (11.8%)	1 (5.9%)	1 (7.1%)
Renal dysfunction	1 (6.3%)	2 (11.8%)	1 (5.9%)	0
Blood pressure lowering	7 (43.8%)	3 (17.6%)	2 (11.8%)	0
QT prolongation (ECG)	2 (12.5%)	0	1 (5.9%)	0
Thrombocytopenia	2 (12.5%)	0	―	―

## Data Availability

The data presented in this study are available on request from the corresponding author due to privacy restrictions.

## Abbreviations

The following abbreviations are used in this manuscript:

AM	Adrenomedullin
SARS-CoV-2	Severe acute respiratory syndrome coronavirus
COVID-19	Coronavirus disease 2019
MV	Mechanical ventilation
VILI	Ventilator-induced lung injury
IBD	Inflammatory bowel disease
ECMO	Extracorporeal membrane oxygenation
RFT	Respiratory function test
DLco	Carbon monoxide diffusion capacity
AE	Adverse event
FAS	Full primary analysis set
